# Glass-Forming Ability, Mechanical Properties, and Energetic Characteristics of ZrCuNiAlNbHfY Bulk Metallic Glasses

**DOI:** 10.3390/ma17133136

**Published:** 2024-06-26

**Authors:** Xin Yu, Jianbin Li, Kaichuang Zhang, Huijie Zhang, Hao Wang, Yuanhang Fang, Yusong Ma, Zhenxiong Wang, Xinggao Zhang, Xiqiang Gai

**Affiliations:** 1 Chemical Defense Institute, Academy of Military Sciences, Beijing 102205, China; 2 School of Chemical Engineering/Xi’an Key Laboratory of Special Energy Materials, Northwest University, Xi’an 710069, China; 3 School of Electromechanical Engineering, North University of China, Taiyuan 030051, China

**Keywords:** Zr-based bulk metallic glasses, glass-forming ability, mechanical properties, energetic characteristics

## Abstract

The effects of partially substituting Al for Cu in Zr_59.62_Cu_18.4-*x*_Ni_12_Al_6+*x*_Nb_3_Hf_0.78_Y_0.2_ (*x* = 0, 2, 4, 6, 8 at.%) bulk metallic glasses (BMGs) on their glass-forming ability (GFA), quasi-static and dynamic mechanical properties, and energy characteristics were investigated. The results showed that an appropriate substitution of Al for Cu can improve GFA and reach a critical casting size up to 10 mm. Additionally, with Al replacement of Cu, the change in the distribution and content of free volume inside the BMGs was the main reason for the quasi-static compression plasticity. In contrast, the BMGs exhibited no plasticity during dynamic compression and high-speed impact, owing to the short loading time and thermal softening effect. In terms of energy characteristics, all alloys have a high combustion enthalpy. And on the surface of the fragments collected from impact, the active elements Zr, Al, and Nb reacted because of the adiabatic temperature rise. Further, *x* = 4 at.% Zr-based BMG with its superior overall performance could penetrate a 6 mm Q235 plate at a speed of 1038 m/s, combining excellent mechanical properties and energy characteristics. This study contributes to the development of Zr-based BMGs as novel energetic structural materials.

## 1. Introduction

Bulk metallic glasses (BMGs) are characterized by high strength, high hardness, large elastic strain limit, and excellent corrosion resistance [1,2,3] due to their unique long-range disordered atomic arrangement structure. And BMGs have a higher free energy and energy-release efficiency than crystalline materials [4,5]. Furthermore, since Zr combines a low activation threshold and high calorific value for oxidation reactions, Zr-based BMGs exhibit a dual destructive effect of penetration and deflagration through energy release on targets, which facilitates military applications such as fragmentation warheads and shaped charges [6,7]. However, because of strain softening and the lack of inherent propagation barriers, shear bands may rapidly crack, leading to catastrophic failure at room temperature [8,9]. Besides plasticity, the practical applications of BMGs are also limited by their poor GFA. Moreover, current energy research has mainly focused on the classic components Zr_55_Ni_5_Al_10_Cu_30_ and Zr_41.25_Ti_13.75_Cu_12.5_Ni_10_Be_22.5_ [7,10,11,12,13], and more BMG systems that simultaneously meet the dimensional, mechanical, and energy requirements are required.

According to recent reports, plasticity can be improved by employing nanocrystals, phase-separated structures, and the introduction of large amounts of free volume [14,15,16]. The first two strategies are typically limited to quasi-static compression, which can dramatically decrease strength and plasticity with an increasing strain rate [15,17]. For instance, the increase in the in situ crystalline phase content of Zr_55_Ni_5_Al_10_Cu_30_ results in enhanced brittleness of fragments under high-speed impact, which promotes adequate fragmentation and releases energy, but weakens the penetration ability of the fragments into the target plate [18]. Elemental substitution is a simple and effective method for improving GFA and mechanical properties. The substitution of alloying elements can promote structural and chemical heterogeneity and adjust the stability of short-range ordering [19,20,21]. The wide distribution of short-range ordering, especially icosahedral-like clusters, increases the difficulty of atomic diffusion and rearrangement, enhancing the stability of supercooled liquids, which is conducive to the formation of an amorphous state [22]. Furthermore, both the free volume content and stability of short-range ordering change under the influence of necessary differences in atomic size and atomic affinity [23,24]. The free-volume-rich region and density packing region contribute to the formation of shear transformation zones and the hindrance of shear bands, respectively, which affect the plasticity of BMGs [25,26]. As Zhu et al. [27] mentioned, substituting Zr with Hf in the range of 0–1.5 at.% adjusted the short-range order structure and increased the free volume content, which effectively enhanced the plasticity and toughness. The difference in atomic size increases random packing density [28], effectively affecting the inhomogeneity of the internal microstructure and local free volume [29]. Tan et al. [24] replaced Zr with Al to stabilize the short-range ordering and improve the GFA, strength, and plasticity of BMGs. Given that Al has a large negative enthalpy of mixing with common base elements such as Zr, Ni, and Nb, the addition and replacement of Al effectively alter the GFA and mechanical properties of BMGs [30,31,32,33].

Considering the superior atomic affinity of Al (Al has a small negative mixing enthalpy only with Cu: −1 kJ/mol) in the Zr-Cu-Ni-Al-Nb-Hf-Y alloy system, Al has a lower Gibbs energy for oxide formation compared to inactive Cu. In this study, the effects of replacing Cu with Al on the GFA, quasi-static and dynamic mechanical properties, and enthalpy of combustion of Zr_59.62_Cu_18.4-_*_x_*Ni_12_Al_6+_*_x_*Nb_3_Hf_0.78_Y_0.2_ (*x* = 0, 2, 4, 6, 8 at.%) were investigated. In addition, the fragments of samples after ballistic gun experiments and the combustion products after oxygen bomb calorimetry experiments were collected for comparative characterization, complementing the energy-releasing characteristics.

## 2. Materials and Methods

### 2.1. Preparation of Zr_59.62_Cu_18.4-x_Ni_12_Al_6+x_Nb_3_Hf_0.78_Y_0.2_ Alloy

The Zr_59.62_Cu_18.4-_*_x_*Ni_12_Al_6+_*_x_*Nb_3_Hf_0.78_Y_0.2_ (*x* = 0, 2, 4, 6, 8 at.%) alloy ingots were prepared in a vacuum arc furnace (WK-II A, Beijing Physcience Optoelectronics Co., Ltd., Beijing, China) using a Ti-gettered argon atmosphere. The purity of raw material was higher than 99.9 wt% (Zhongnuo New Material Technology Co., Ltd., Beijing, China). The intermediate Zr-Nb alloy was first pre-melted and then mixed with other metals and remelted four times to ensure compositional homogeneity. The surface oxide layer of the ingot was polished. Then, a series of cylindrical rods with diameters (Φ) of 3, 5, 7, 8, 10, and 12 mm and a length (l) of 150 mm was prepared using water-cooled copper-mold suction-casting equipment (ML-MG35, Peshing New Metal Co., Ltd., Changzhou, China). And the melt-spun ribbons were prepared by a melt-spinning machine (VF-RQT50, Makabe Giken Co., Ltd., Sendai, Japan) at a speed of 20 m/s.

### 2.2. Microstructure Characterization

The structures and morphologies of the samples were analyzed at room temperature using X-ray diffraction (XRD, Bruker D8 Advance, Karlsruhe, Germany), scanning electron microscopy (SEM, JSM 7200F, Tokyo, Japan), and high-resolution transmission electron microscopy (HRTEM, FEI Talos F200X, Waltham, MA, USA). The HRTEM sample was cut from the rods with Φ = 3 mm and thinned down to a thickness of 30 µm using SiC papers, and then prepared using a precision ion polishing system (PIPS, Gatan-691, Pleasanton, CA, USA). The thermal parameters were obtained using differential scanning calorimetry (DSC, Netzsch 404F3, Selb, Germany) in a flow argon atmosphere with a heating rate of 20 K/min, and DSC specimens, which had a weight of 10–15 mg, were taken from the same parts of the Φ = 3 mm rods.

### 2.3. Performance Test

The as-cast samples with Φ = 3 mm were machined into a length-to-width ratio of 2:1 for quasi-static compression and 1:1 for dynamic compression. All samples were carefully polished to ensure parallel ends. Quasi-static compression tests were performed using a universal testing machine (MTS E43.504, Eden Prairie, MN, USA) at a strain rate of 1 × 10^−3^ s^−1^, and dynamic compression tests were performed using the Split Hopkinson Pressure Bar (SHPB, ATL1500, Archimedes Industry Technology Co., LTD., Beijing, China) at a strain rate of 3 × 10^3^ s^−1^. Each compression test was repeated three times to ensure the reliability of the data.

A microcomputer automatic oxygen bomb calorimeter (TRHW-7000E, Tianrun Electronic Technology Co., Hebi, China) was used in 3 MPa pure oxygen to measure the combustion enthalpy at room temperature, and the weight of the test samples prepared from the melt-spinning method for each test was about 0.2 g. Ballistic gun (Nanjing University of Science and Technology, Nanjing, China) tests were used to investigate the damage performance of the BMG samples with Φ = 10 mm and l = 10 mm. The targets were two separate plates placed 1000 mm apart, including a 6 mm thick Q235 plate and a 1 mm thick aftereffect Al plate, to observe the fragmentation distribution of the fragments. The penetration process was recorded using a high-speed camera (Phantom, Wayne, NJ, USA). The fragments generated under high-speed impact were collected in a closed steel chamber.

## 3. Results and Discussion

### 3.1. GFA and Thermal Ability

**Figure 1:**
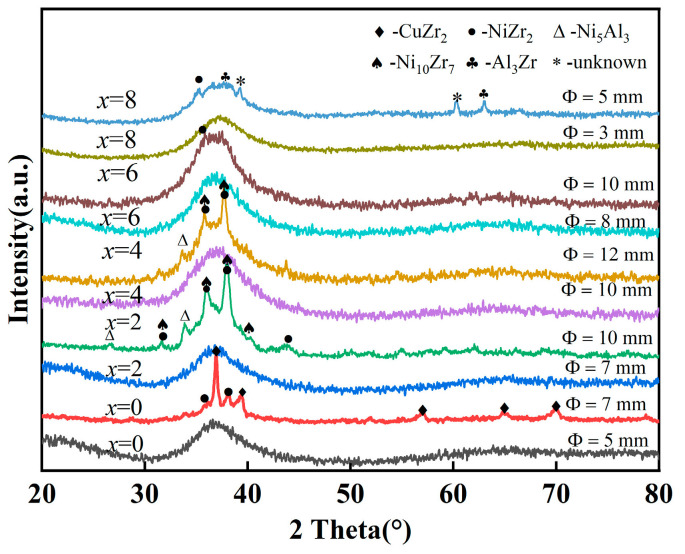
XRD patterns of Zr_59.62_Cu_18.4-*x*_Ni_12_Al_6+*x*_Nb_3_Hf_0.78_Y_0.2_ (*x* = 0, 2, 4, 6, 8 at.%) BMGs with different diameters (Φ).

Figure 1 shows the XRD patterns of as-cast Zr_59.62_Cu_18.4-_*_x_*Ni_12_Al_6+_*_x_*Nb_3_Hf_0.78_Y_0.2_ (*x* = 0, 2, 4, 6, 8 at.%) alloy rods with different diameters. The XRD patterns under critical diameter (*D*_c_) conditions exhibit a broad diffraction peak, indicating that the cast samples are completely amorphous within the resolution range of the XRD technique. When Al replaces Cu, the *D*_c_ of the BMGs increases at first and then decreases. *D*_c_ reaches a maximum of more than 10 mm at *x* = 4 at.% and approaches 10 mm at *x* = 6 at.%, which is more than twice that of *x* = 8 at.%. When the diameter exceeds the *D*_c_, many crystalline phases precipitate.

**Figure 2:**
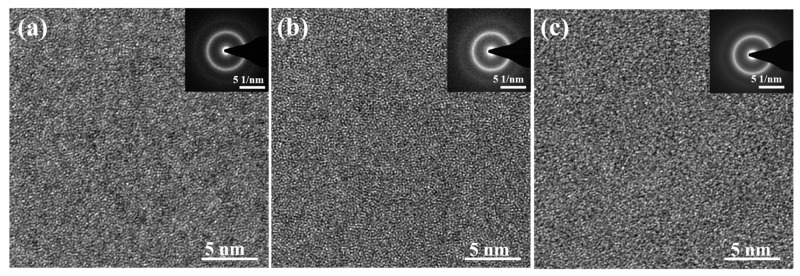
The HRTEM and corresponding SAED (in the inset) images of the prepared Zr_59.62_Cu_18.4-*x*_Ni_12_Al_6+*x*_Nb_3_Hf_0.78_Y_0.2_ (*x* = 0, 4, 8 at.%) BMGs with Φ = 3 mm: (**a**) *x* = 0; (**b**) *x* = 4; (**c**) *x* = 8.

The HRTEM image and corresponding selected area electron diffraction pattern (SAED) of *x* = 0, *x* = 4, and *x* = 8 at.% BMGs with Φ = 3 mm are shown in Figure 2. The results show typical amorphous structures with isotropic maze patterns and the SAED in the inset displays a typical amorphous halo ring with no diffraction spots, indicating the glassy nature of the rod.

**Figure 3:**
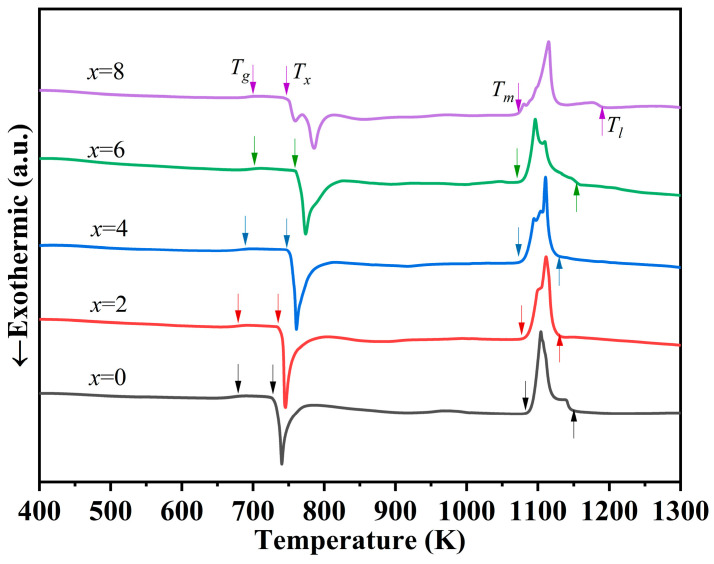
DSC curves of Zr_59.62_Cu_18.4-*x*_Ni_12_Al_6+*x*_Nb_3_Hf_0.78_Y_0.2_ (*x* = 0, 2, 4, 6, 8 at.%) BMGs with Φ = 3 mm at heating rate of 20 K/min. (*T_g_*: the glass transition temperature; *T_x_*: the onset temperature of crystallization; *T_m_*: the melting temperature; *T_l_*: the liquids temperature).

The DSC curves of Zr_59.62_Cu_18.4-_*_x_*Ni_12_Al_6+_*_x_*Nb_3_Hf_0.78_Y_0.2_ (*x* = 0, 2, 4, 6, 8 at.%) BMGs with Φ = 3 mm at a heating rate of 20 K/min are shown in Figure 3. In the temperature range of 400–1300 K, some evident glass transition characteristics and the production of exothermic peaks corresponding with crystallization are observed. A two-stage crystallization exothermic peak appears at *x* = 8 at.%, suggesting that Al substitution changes the internal structure of BMGs and affects the crystallization process. In Zr-Cu-Ni-Al-Nb and many other multicomponent BMGs, the formation of crystal phases can be thermodynamically divided into several stages [20,24,26,27]. The first stage is the precipitation of primary crystal phases from supercooled liquids, which mainly refers to the precipitation of local ordering clusters. The other stages correspond to the formation of stable crystal phases. When the instability of the precipitated local ordering clusters gradually increases upon the Al content exceeding (x = 8 at.%), the corresponding precipitation crystallization temperature decreases, resulting in a single crystallization event divided into two steps, which is similar to the research of Li and Tan, et al. [24,34].

The detailed thermal parameters are listed in Table 1. When Cu is gradually substituted by Al, both the glass transition temperature (*T_g_*) and the onset temperature of crystallization (*T_x_*) initially increase before decreasing. The supercooled liquid region (Δ*T* = *T_x_* − *T_g_*) obtains a maximum value of 59 K at *x* = 4 at.%. Inoue et al. [2] reported that a larger Δ*T* could indicate a more stable supercooled liquid. Two other parameters reflecting GFA, namely reduced glass transition temperature (*T_rg_* = *T_g_*/*T_l_*) and the γ parameter (γ = *T_x_*/*(T_g_* + *T_l_*)), also exhibit the largest values of 0.610 and 0.411, respectively, indicating that *x* = 4 at.% BMG has the best GFA and stability in the system. Moreover, as the Al content increases, the liquids temperature (*T_l_*) decreases and then increases, and the *x* = 4 at.% alloy exhibits the lowest *T_l_* of 1130 K, reflecting that this composition is closer to the eutectic point when compared with others. The thermodynamic stability of the molten liquid phase is higher when the *T_l_* of deep eutectic composition is lower. In other words, the *x* = 4 at.% alloy possesses the highest stability of alloy melting against crystallization upon cooling and ultimately achieves the highest GFA.

In a multicomponent system, it is known that the mixed enthalpy (Δ*H_i-j_*) and atomic size are the two main factors affecting GFA. Cu has a positive Δ*H_i-j_* with major elements Ni (∆*H_Cu-Nb_* = 3 kJ/mol) and Nb (∆*H_Cu-Ni_* = 4 kJ/mol), and excess Cu may weaken the strong bonding structure between atoms and reduce the packing densities of atoms. In contrast, Al has a large negative Δ*H_i-j_* with all other elements Zr, Ni, Nb, Y, and Hf (Δ*H_Al-__Zr_* = −44 kJ/mol, Δ*H_Al-Cu_* = −1 kJ/mol, Δ*H_Al-Ni_* = −22 kJ/mol, Δ*H_Al-N__b_* = −18 kJ/mol, Δ*H_Al-__Hf_* = −38 kJ/mol, Δ*H_Al-__Y_* = −39 kJ/mol), preferring to form atomic pairs. The system mixing enthalpy (Δ*H_mix_*) of the Zr-Cu-Ni-Al-Nb-Y-Hf system can be calculated using the following equation [35]:

(1) $${∆H}_{mix}=\sum_{i\neq j}4{{∆H}_{i-j}x}_{i}x_{j}$$

where *x_i_* and *x_j_* are the atomic percentages of the elements i and j, respectively, and the values of ${∆H}_{i-j}$ are obtained from the literature [36]. As summarized in Table 1, the Δ*H_mix_* of the system increases when Cu is substituted by Al. An enhanced negative Δ*H_mix_* would encourage the chemical short-range order. On the other hand, in terms of atomic size, appropriate differences in atomic size increase the random packing density and improves GFA [37]. However, when the Al content continues to increase (*x* = 8 at.%), some cluster structures are unstable and preferentially precipitate, resulting in a decrease in *T_g_* and the degradation of GFA.

### 3.2. Quasi-Static and Dynamic Mechanical Properties of BMGs

**Figure 4:**
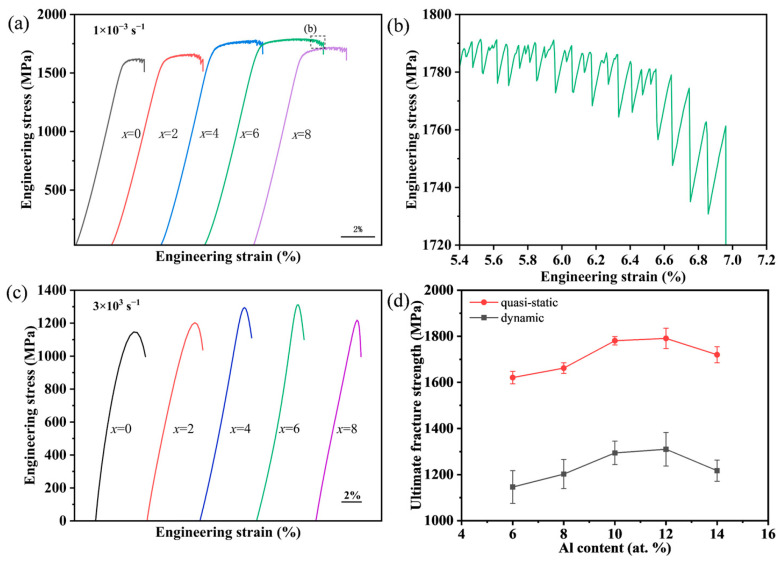
The mechanical behavior of BMGs: (**a**) quasi-static compressive stress–strain curves of Zr_59.62_Cu_18.4-*x*_Ni_12_Al_6+*x*_Nb_3_Hf_0.78_Y_0.2_ (*x* = 0, 2, 4, 6, 8 at.%) BMGs at a strain rate of 1 × 10^−3^ s^−1^ and the serrated flow behaviors in region b of the *x* = 6 curve; (**b**) the magnified image of serrated flow behaviors in (**a**); (**c**) dynamic compressive stress–strain curves of BMGs at a strain rate of 3 × 10^3^ s^−1^; (**d**) the quasi-static and dynamic fracture strength of the as-cast specimens as a function of Al content.

The stress–strain curves for the quasi-static and dynamic compression of the BMGs at strain rates of 1 × 10^−3^ s^−1^ and 3 × 10^3^ s^−1^ are shown in Figure 4a,c. Characterization data obtained from the curves are listed in Table 2, including yield strength (σ_y_), fracture strength (σ_max_), and plastic strain (ε_p_). Under quasi-static compressive loading, the σ_y_ value of the *x* = 0 at.% alloy is 1582 MPa and the ε_p_ value is 1.06%. When the Al content increases from *x* = 0 at.% to *x* = 6 at.%, both σ_y_ and ε_p_ of the alloys increase and then decrease with the increasing Al content. The *x* = 6 at.% alloy exhibits the largest σ_y_ of 1722 MPa and a ε_p_ of 3.45%, which is 3.25 times larger than that of the pristine alloy. While there exist serrated flow behaviors (Figure 4b) in quasi-static compressive deformation, reflecting that a large number of shear bands were formed to adapt to the plastic strain, the dynamic fracture is complete elastic deformation without any macroplasticity. As shown in Figure 4d, when Al replaces Cu, the quasi-static and dynamic compression fracture strengths follow basically the same trend. And the compressive strength decreases as the strain rate increases, and the dynamic fracture strength ranges from 1100 to 1310 MPa.

**Figure 5:**
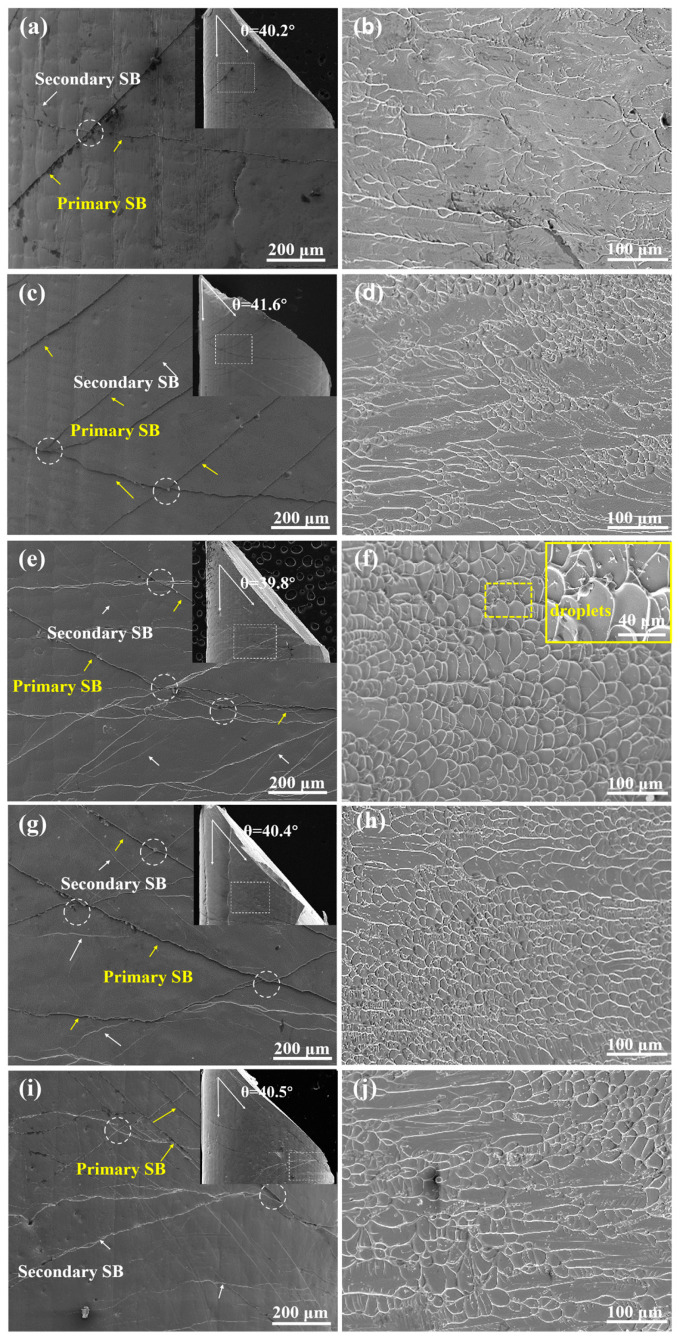
The lateral and fracture surfaces of Zr_59.62_Cu_18.4-*x*_Ni_12_Al_6+*x*_Nb_3_Hf_0.78_Y_0.2_ (*x* = 0, 2, 4, 6, 8 at.%) BMGs after quasi-static compression: (**a**,**b**) *x* = 0; (**c**,**d**) *x* = 2; (**e**,**f**) *x* = 4; (**g**,**h**) *x* = 6; (**i**,**j**) *x* = 8. (Yellow arrows: the primary shear bands; White arrows: the secondary shear band; Circles: bifurcations and crisscrossings of shear bands; Yellow region: the molten droplets and its magnified image).

To further clarify the deformation mechanism of BMGs, Figure 5 shows the lateral and fracture surface morphology images after the final fracture. The fracture surface angle is found to be from 39.8° to 41.7°. Under compression, the normal stresses prohibit the shear fracture of the BMG, resulting in a fracture angle of less than 45° [38,39]. Some primary and secondary shear bands (SBs) appear on the specimens, and the numbers of SBs and their bifurcations and crisscrossings (marked by circles) at *x* = 4 and 6 at.% are higher than those at *x* = 0, 2, and 8 at.%. In the fractography images, the specimens with *x* = 4 and 6 at.% exhibit more vein-like patterns along the direction of shear stress, caused by the instantaneous release of high elastic energy within the shear bands, while the *x* = 0 at.% BMG demonstrates the largest proportion of smooth areas. The plasticity is revealed by the interaction between SBs and the competitive relationship between the patterned regions and smooth regions. The high density of shear bands and vein patterns generally reflects good plasticity [14,40,41].

As mentioned above, Al has a large negative ΔH_i-j_ with other elements. In particular, Al and Zr easily form stable Al-Zr atomic pairs because they have the largest negative enthalpy of mixing, which may contribute to their strength in a certain range [36]. In this system, the atomic radii of Zr, Cu, Ni, Al, Nb, Hf, and Y are 0.162, 0.128, 0.125, 0.143, 0.143, 0.159, and 0.181 nm, respectively. The proper replacement of small atoms of Cu with Al may lead to enhanced structural inhomogeneity and an increase in free volume. According to the free-volume model, the loosely packed defective regions with a higher free volume serve as the initiation regions for shear band generation during plastic deformation, while the high-density-packing area usually inhibits the propagation of the SBs [20], causing the SBs to proliferate, branch, and deflect. In such interactions, the formation of numerous shear bands is promoted, resulting in greater plasticity.

**Figure 6:**
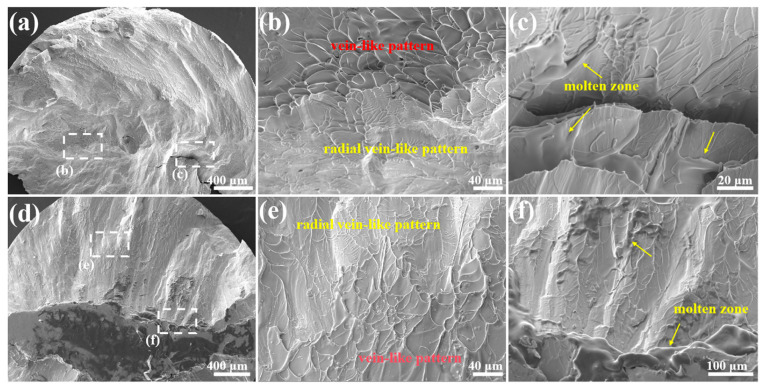
The fracture surfaces of Zr_59.62_Cu_18.4-*x*_Ni_12_Al_6+*x*_Nb_3_Hf_0.78_Y_0.2_ (*x* = 4, 8 at.%) BMGs after dynamic compression: (**a**) the full view of *x* = 4 BMG fracture surface and the classic patterns (**b**) and molten zone (**c**); (**b**) the magnified image of radial vein-like pattern and vein-like pattern of *x* = 4 BMG; (**c**) the magnified image of molten zone of *x* = 4 BMG; (**d**) the full view of *x* = 8 BMG fracture surface and the classic patterns (**e**) and molten zone (**f**); (**e**) the magnified image of radial vein-like pattern and vein-like pattern of *x* = 8 BMG; (**f**) the magnified image of molten zone of *x* = 8 BMG.

Contrary to the vein-like patterns with obvious density differences under quasi-static compression, the fracture surfaces under dynamic compression show a complex pattern with no obvious regularity. Figure 6 shows the typical dynamic fracture surfaces of the *x* = 4 at.% alloy and the *x* = 8 at.% alloy. In addition to vein-like patterns, there are dendrite-like patterns, cracks, and molten zones. The direct linking of the vein-like pattern and the radial vein-like pattern indicates a direct transition from compressive shear stress into tensile shear stress [42], as shown in Figure 6b,e. Moreover, the molten droplets produced during quasi-static compression are approximately 10 µm (Figure 5f), and the size of the molten zones at the edge of the cracks after dynamic compression can reach more than 200 µm (Figure 6c,f), which indicates that the temperature rise caused by adiabatic shearing exceeds the glass transition temperature or even the melting point of the alloys during quasi-static compression [43], and is greater under dynamic compression.

Compared with quasi-static compression, the high-density-packing area does not prohibit the propagation of SBs [44], and there is not enough time for shear transformation zones to adapt to the dynamic strain rate, resulting in premature failure of the samples. The free-volume softening and thermal softening effects for dynamic compression are more remarkable than those for quasi-static compression [45]; thus, the dynamic strength is lower than that under quasi-static loading. It should be emphasized that despite the overall brittle morphology, local toughness persists as vein-like patterns (Figure 6b,e) under dynamic compression.

### 3.3. Energetic Characteristics

**Figure 7:**
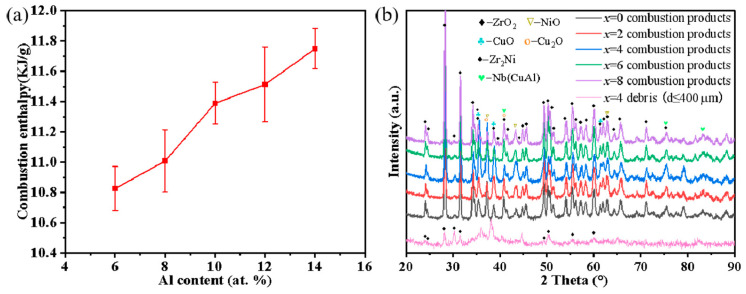
(**a**) Average mass combustion enthalpy of Zr_59.62_Cu_18.4-*x*_Ni_12_Al_6+*x*_Nb_3_Hf_0.78_Y_0.2_ (*x* = 0, 2, 4, 6, 8 at.%); (**b**) XRD patterns of combustion products of Zr_59.62_Cu_18.4-*x*_Ni_12_Al_6+*x*_Nb_3_Hf_0.78_Y_0.2_ (*x* = 0, 2, 4, 6, 8 at.%) and recovered impact debris of x = 4 at.% BMG fragment.

The average mass combustion enthalpy of the Zr_59.62_Cu_18.4-_*_x_*Ni_12_Al_6+_*_x_*Nb_3_Hf_0.78_Y_0.2_ (*x* = 0, 2, 4, 6, 8 at.%) melt-spun ribbon measured by the oxygen bomb calorimeter is above 10 kJ/g (TNT: 4.1 kJ/g). As the Al content increases from 6 at.% to 14 at.%, the combustion enthalpy of the alloys has a slightly increasing trend (Figure 7a), from 10.826 KJ/g at *x* = 0 at.% to 11.749 KJ/g at *x* = 8 at.%. Hu et al. [46] have reported that the average mass combustion enthalpy of Zr_55_Cu_30_Ni_5_Al_10_ and Zr_41.2_Ti_13.8_Cu_12.5_Ni_10_Be_22.5_ is 10.797 KJ/g and 6.496 KJ/g, respectively. Our results show that the Zr_59.62_Cu_18.4-_*_x_*Ni_12_Al_6+_*_x_*Nb_3_Hf_0.78_Y_0.2_ alloy has a higher combustion enthalpy. And the main combustion products are ZrO_2_ and a small amount of CuO, Cu_2_O, and NiO (Figure 7b).

**Figure 8:**
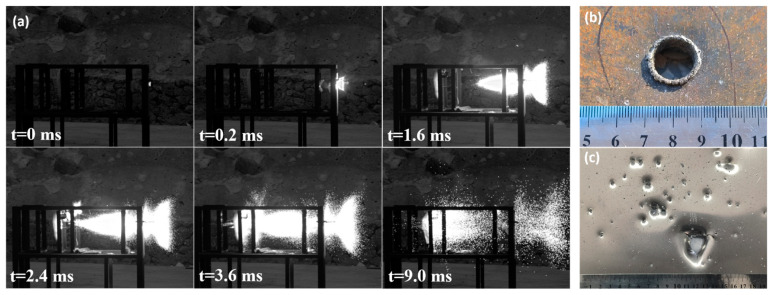
The x = 4 at.% Zr-based BMG fragment penetrating two separated plates at a speed of 1038 m/s: (**a**) the penetration process; (**b**) the holes formed on the Q235 target; (**c**) the distribution of debris on the Al target.

Among all of the alloys, the *x* = 4 at.% BMG demonstrates excellent comprehensive properties, so it is necessary to verify its target penetration capability. The high-speed photography images of the *x* = 4 at.% BMG passing through the targets at a speed of 1038 m/s are shown in Figure 8. Under impact, the BMG firstly penetrates the Q235 target, leaving a hole larger than 10 mm and melting traces around it caused by the violent reaction during the penetration process. The point to emphasize is that no burning is observed before the BMG hits the target, implying that it does not crack prematurely under detonation loading. The remaining fragments subsequently penetrate and damage the Al plate with kinetic and chemical energy. The light emitted by impacts arises from burning debris [47]. Due to the large specific surface area, small fragments are fully in contact with oxygen and contribute more to the chemical reaction, releasing energy to generate flames and leaving black reaction traces on the Al plate. In the recovered impact debris (dimensions less than 400 μm), only the characteristic peaks of ZrO_2_ are observed and other oxides are contained insufficiently to be detected (Figure 7b).

**Figure 9:**
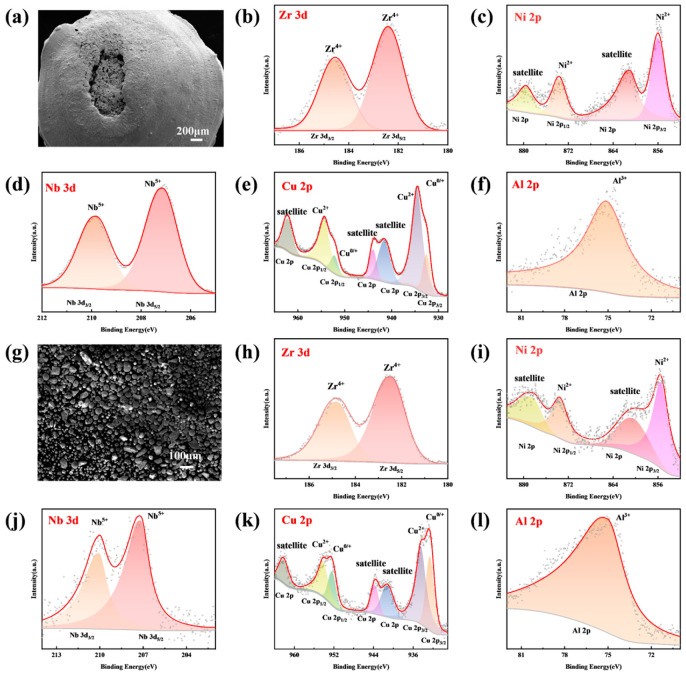
SEM images of samples and XPS results of Zr, Cu, Ni, Al, and Nb elements on surface of (**a**–**f**) combustion products of x = 4 at.% alloy; (**g**–**l**) recovered impact debris of x = 4 at.% alloy.

In order to confirm the oxidation state of the constituent elements, XPS analysis is performed. As shown in Figure 9, on the surface of the combustion products and recovered impact debris, all elements show oxidation peaks, indicating that the elements all participate in the oxidation reaction. Zr, Ni, Al, and Nb elements are fully oxidized, and Cu is relatively inert because of the existence of partially incompletely oxidized Cu. By comparison, the oxidation degree of Cu in the combustion products is higher than that in the impact debris.

**Figure 10:**
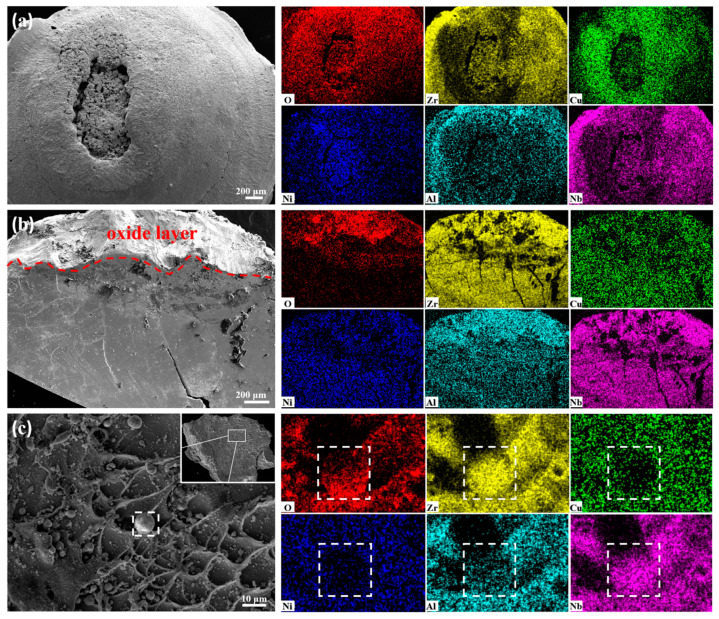
SEM and EDS images: (**a**) the combustion products of the x = 4 at.% alloy; (**b**) the oxide layer in the cross-section of the impact debris of the x = 4 at.% alloy; (**c**) the hollow spherical particles (the marked box area) on the surface of the impact debris of the x = 4 at.% alloy.

To further understand the mechanism of energy release, SEM and EDS are used to analyze the combustion products, the oxide layer on the cross-section of the impact debris, and the hollow spherical particles on the surface of the impact debris, as shown in Figure 10. Obviously, there are clearly distinguished Zr-rich phases and Cu-rich phases in the combustion products (Figure 10a), which are caused by the uneven distribution of oxides produced by complete combustion. Moreover, in the oxide layer (Figure 10b), the active elements Zr, Al, and Nb demonstrate obvious concentrated distribution with O, and the relative distributions of Cu and Ni are different. Segregation does not exist in as-cast amorphous alloys, and because of the higher melting point of Nb than that of Cu, this phenomenon of differing distributions is mainly caused by the preferential combustion of active elements rather than just simple melting, which becomes more pronounced with an increase in the degree of oxidation reaction. According to their Gibbs energies of formation (per mole of oxygen) [48], the order of elements that are more likely to react with O is Zr > Al > Nb > Ni > Cu. Under high-speed impact, the smaller the size of the fragments, the higher the degree of oxidation [49]; so, the separation is more severe in hollow spherical particles (Figure 10c) than in the oxide layer.

## 4. Conclusions

We have reported the GFA, mechanical performance, and energetic characteristics of Be-free Zr_59.62_Cu_18.4-*x*_Ni_12_Al_6+*x*_Nb_3_Hf_0.78_Y_0.2_ (*x* = 0, 2, 4, 6, 8 at.%) BMGs. With increasing Al content, the GFA increases significantly and reaches a maximum *D*_c_ of 10 mm at *x* = 4 at.%. And the *x* = 6 at.% BMG possesses the highest σ_max_ of 1791 MPa and the best quasi-static compression plasticity of 3.45%. In contrast, owing to the short loading time and thermal softening effect, no plasticity exists in Zr-based BMGs during high-rate compression. Regarding the energy characteristics, the increase in Al content increases the enthalpy of combustion from 10.826 KJ/g at *x* = 0 at.% to 11.749 KJ/g at *x* = 8 at.%. And elements Zr, Al, and Nb burn preferentially over Cu and Ni under impact due to different oxygen affinities on the surface of the fragments collected from the impact. The *x* = 4 at.% Zr-based BMG with its superior overall performance could penetrate a 6 mm Q235 plate at a speed of 1038 m/s. In conclusion, Zr-based BMGs exhibit a superior enthalpy of combustion, high-energy release efficiency, and penetration ability of targets, which allows them to be applied as energetic fragments, a matrix alloy in armor-piercing penetrators, and linear-shaped charges to achieve post-target damage effects.

## Figures and Tables

**Table 1:** *D*_c_ and thermal parameters of Zr_59.62_Cu_18.4-_*_x_*Ni_12_Al_6+_*_x_*Nb_3_Hf_0.78_Y_0.2_ (*x* = 0, 2, 4, 6, 8 at.%).

Alloys	*D*_c_ (mm)	*T*_g_ (K)	*T_x_* (K)	*T_m_* (K)	*T_l_* (K)	Δ*T* (K)	*T_rg_*	γ	*∆H_mi_*_x_ (kJ/mol)
*x* = 0	5	679	728	1080	1150	49	0.590	0.398	−31.3
*x* = 2	7	682	735	1076	1132	53	0.602	0.405	−32.6
*x* = 4	10	689	748	1072	1130	59	0.610	0.411	−34.0
*x* = 6	8	702	758	1071	1154	56	0.608	0.408	−35.3
*x* = 8	3	700	747	1069	1190	47	0.588	0.395	−36.6

**Table 2:** Quasi-static and dynamic compressive parameters of Zr_59.62_Cu_18.4-_*_x_*Ni_12_Al_6+_*_x_*Nb_3_Hf_0.78_Y_0.2_ (*x* = 0, 2, 4, 6, 8 at.%).

Alloys	$\dot{\varepsilon }$ (s^−1^)	$\sigma_{y}$ (MPa)	$\sigma_{max}$ (MPa)	$\varepsilon_{p}$ (%)
*x* = 0	1 × 10^−3^	1582 ± 31	1621 ± 27	1.06 ± 0.31
	3 × 10^3^	-	1146 ± 71	-
*x* = 2	1 × 10^−3^	1610 ± 27	1662 ± 23	1.95 ± 0.33
	3 × 10^3^	-	1202 ± 63	-
*x* = 4	1 × 10^−3^	1707 ± 19	1781 ± 18	2.68 ± 0.17
	3 × 10^3^	-	1294 ± 51	-
*x* = 6	1 × 10^−3^	1722 ± 36	1791 ± 44	3.45 ± 0.21
	3 × 10^3^	-	1310 ± 73	-
*x* = 8	1 × 10^−3^	1658 ± 33	1720 ± 35	2.16 ± 0.27
	3 × 10^3^	-	1217 ± 46	-

## Data Availability

The data presented in this study are available on request from the corresponding author.
